# Dietary Protein Source Mediates Colitis Pathogenesis Through Bacterial Modulation of Bile Acids

**DOI:** 10.1016/j.jcmgh.2026.101825

**Published:** 2026-06-24

**Authors:** Simon M. Gray, Michael C. Wood, Samantha C. Mulkeen, Sunjida Ahmed, Shrey D. Thaker, Bo Chen, William R. Sander, Vladimira Bibeva, Xiaoyue Zhang, Jie Yang, Jeremy W. Herzog, Anh D. Moss, Shiying Zhang, Belgin Dogan, Kenneth W. Simpson, R. Balfour Sartor, David C. Montrose

**Affiliations:** 1 Department of Medicine, Center for Gastrointestinal Biology and Disease, University of North Carolina at Chapel Hill, Chapel Hill, North Carolina; 2 Department of Microbiology and Immunology, University of North Carolina at Chapel Hill, Chapel Hill, North Carolina; 3 National Gnotobiotic Rodent Resource Center, University of North Carolina at Chapel Hill, Chapel Hill, North Carolina; 4 Department of Pathology, Renaissance School of Medicine, Stony Brook University, Stony Brook, New York; 5 Biostatistical Consulting Core, Renaissance School of Medicine, Stony Brook University, Stony Brook, New York; 6 Department of Family, Population and Preventive Medicine, Stony Brook University, Stony Brook, New York; 7 Department of Nutrition, University of North Carolina at Chapel Hill, Chapel Hill, North Carolina; 8 Department of Clinical Sciences, Cornell University, Ithaca, New York; 9 Stony Brook Cancer Center, Stony Brook, New York

**Keywords:** Dextran Sodium Sulfate, Experimental Colitis, Interleukin10 Deficient Mice, Microbiome

## Abstract

**Background & Aims:**

Evidence-based dietary recommendations for individuals with inflammatory bowel diseases are limited. Red meat consumption is associated with increased inflammatory bowel disease incidence and relapse in patients, suggesting that switching to a plant-based diet may limit gut inflammation. However, the components and mechanisms underlying the differential effects of these diets remain poorly understood. This study investigated the differential impact of dietary protein source on experimental colitis and related mechanisms.

**Methods:**

Isocaloric synthetic diets containing protein isolates from beef, egg whites, casein, soy, or pea were tested in acute (dextran sodium sulfate–induced) and chronic (*Il10-*deficient) murine colitis models. Gut resident microbes were quantified with 16S-sequencing, and their role was evaluated by antibiotic depletion, germ-free, selectively colonized gnotobiotic, and fecal microbiota transplant mouse studies.

**Results:**

Mice fed a beef protein diet exhibited the most severe colitis, whereas mice fed pea protein developed mild inflammation. The colitis-promoting effects of beef protein were microbially mediated. In the absence of colitis, beef protein feeding reduced the abundance of *Lactobacillus johnsonii* and *Turicibacter sanguinis* and expanded *Akkermansia muciniphila*, which localized to the mucus in association with decreased mucus thickness and quality. Beef protein–fed mice had elevated primary and conjugated fecal bile acids, and taurocholic acid administration to pea protein–fed mice worsened colitis. Dietary psyllium protected against beef protein–mediated inflammation, restored bile acid–modulating commensals, and normalized bile acid ratios.

**Conclusions:**

These data suggest that the protein component of red meat may be responsible, in part, for the colitis-promoting effects of this food source and provide insight into dietary factors that may influence inflammatory bowel disease severity.

**What You Need to Know**

**Background**

Although diet is believed to be an important contributor to the pathogenesis of inflammatory bowel diseases, there are few evidence-based dietary recommendations for patients to lessen disease severity.

**Impact**

The findings in this study suggest that the protein component of plant vs animal-based diets is a mediator of the beneficial vs harmful effects of each diet type in colitis.

**Future Directions**

These findings provide a rationale for determining whether modifying dietary protein source could lessen the severity of disease in patients with inflammatory bowel diseases.

Diet influences the pathogenesis of inflammatory bowel diseases (IBD), disorders of chronic intestinal inflammation caused by dysregulated host immune responses to resident intestinal microbes.^1^, ^2^, ^3^ Despite this knowledge, limited evidence-based dietary recommendations exist to reduce the incidence and severity of IBD.^4^, ^5^, ^6^ IBD incidence has increased in the United States and globally over multiple decades, including in regions with previously low rates.^7^, ^8^, ^9^ This increase has occurred in parallel with elevated consumption of animal-based protein, including red meat, suggesting a link between animal protein consumption and IBD.^10^^,^^11^ Correspondingly, prospective and retrospective human cohort studies have associated animal protein and red meat intake with increased risk of incident and relapsed IBD.^12^, ^13^, ^14^, ^15^ However, the mechanisms by which specific dietary protein sources mediate gut inflammation remain unclear.

Diet regulates gut homeostasis and inflammation, in part, by shaping the composition, function, and metabolic activity of the gut microbiota.^2^^,^^16^, ^17^, ^18^, ^19^, ^20^, ^21^, ^22^, ^23^, ^24^, ^25^ For example, reducing dietary fiber or increasing fructose consumption in mice enriches mucus-digesting resident bacteria, such as *Akkermansia muciniphila*.^16^^,^^21^, ^22^, ^23^^,^^25^ Further, consuming high amounts of fructose or milk fat limits the deconjugation of bile acids (BAs) (small molecules synthesized by the liver and modified by intestinal microbes), resulting in increased levels of conjugated BAs (CBAs) and exacerbated experimental colitis.^21^^,^^26^ Notably, patients with active IBDs have higher levels of primary and CBAs and depleted secondary unconjugated BAs, relative to healthy controls.^27^, ^28^, ^29^ Moreover, primary CBAs, which are metabolized by microbial enzymes to unconjugated secondary BAs, exacerbate inflammation in experimental colitis models.^26^^,^^28^^,^^30^ Modification of luminal intestinal bile acid species is dependent on the prevalence and activity of bile salt hydrolase and bile-acid 7α-dehydroxylase enzymes in the gut microbiome, which can be altered by antibiotics, inflammation, and diet.^26^^,^^30^^,^^31^ However, the role of dietary protein source in these complex interactions and development of colonic inflammation is poorly understood.

In this study, we demonstrate that dietary protein source is a critical mediator of colitis severity across multiple murine models of IBD. The differences in inflammation across diet groups are driven by the capacity of microbes to modify the gut barrier and modulate BAs. Collectively, these findings reveal a novel role for the protein component of diet in modulating colitis pathogenesis. These clinically relevant results have the potential to guide dietary recommendations for patients with IBD, with the goal of reducing disease incidence and severity.

## Results

### Dietary Protein Source Mediates Colitis Severity

**Figure 1:**
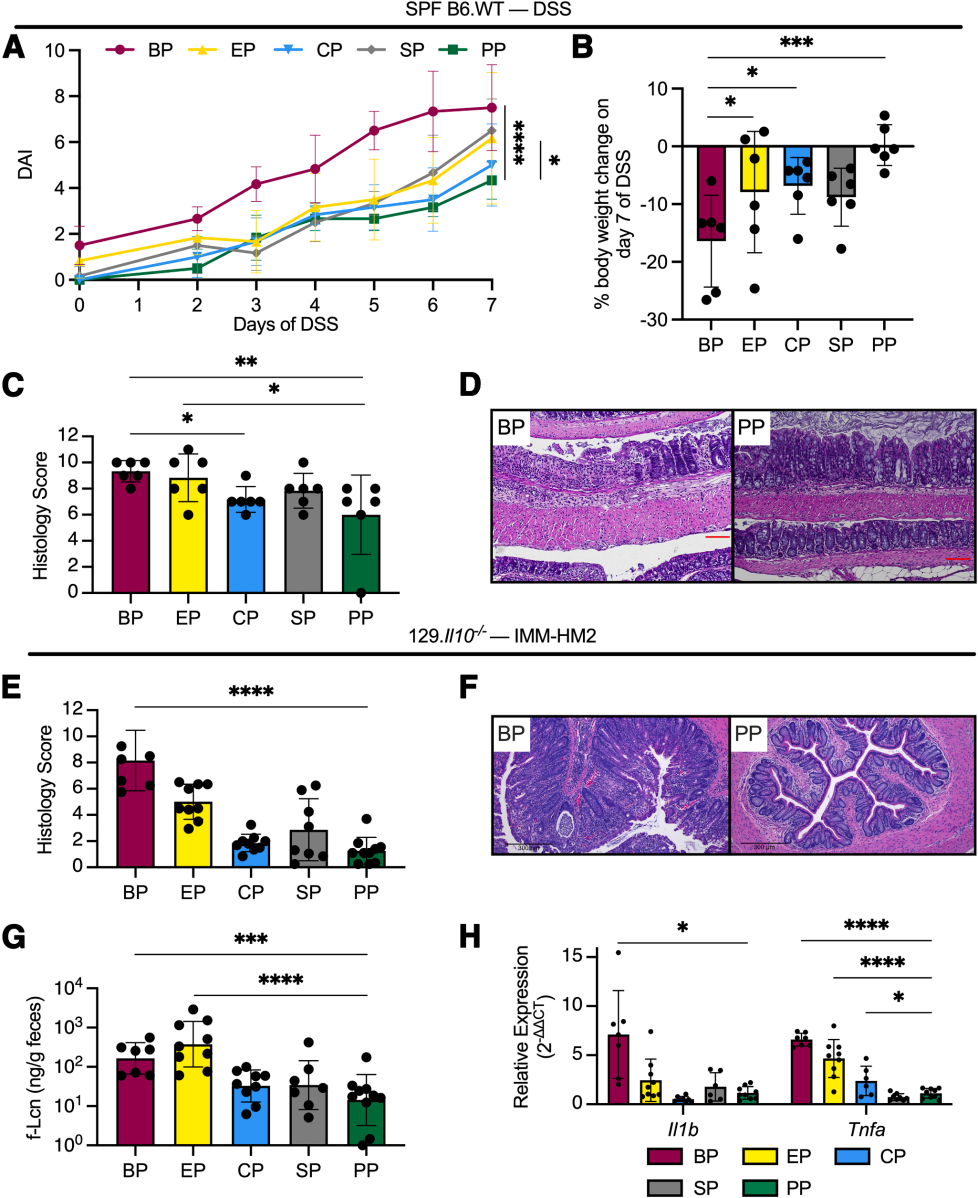
**Dietary BP worsens murine colitis compared with other protein sources.** (*A–D*) C57BL/6 mice were fed isocaloric diets containing protein isolate derived from beef (BP), egg whites (EP), casein (CP), soy (SP), or pea (PP) for 7 days, followed by administration of 1% DSS for 7 days, while being continued on the respective diets. The DAI was measured during DSS exposure (*A*), percent of body weight change relative to day 0 of DSS exposure was calculated following 7 days of DSS administration (*B*), and the histologic score was assessed in colons on day 7 of DSS administration (*C*) (n = 6 mice per group), with representative histologic images of colons from BP or PP-fed mice shown (*D*). (*E–H*) GF 129S6/SvEv *Il10*^*−/−*^ mice were inoculated with MA pooled human IBD patient fecal microbiota (IMM-HM2) then fed the diets described in (*A*) for 14 days. Total histology score was assessed (*E*) with representative histologic images of colons shown (*F*), fecal lipocalin-2 (f-Lcn) levels were measured (*G*), and relative gene expression of cytokines was measured in colons (*H*), on day 14. n = 7–10 mice per group. Data are pooled from 2 independent experiments. ∗*P* < .05; ∗∗*P* < .01; ∗∗∗*P* < .001; ∗∗∗∗*P* < .0001. Significance indicators for (*A*) represent differences in values over 7 days. Bar plots show mean and standard deviation.

To investigate the impact of dietary protein source on experimental colitis severity, 5 isocaloric synthetic diets containing protein isolates from beef (BP), egg whites, casein, soy or pea (PP) were tested in acute and chronic murine colitis models (Table 1). Testing multiple colitis models driven by distinct immunologic pathways and pathologic features increases confidence in and clinical applicability of observed phenotypes. First, these diets were fed to male wild-type (WT) specific pathogen–free (SPF) C57BL/6J mice during 1% dextran sodium sulfate (DSS)-mediated induction of colitis. Mice fed a BP diet developed the most severe colitis, whereas those fed a PP diet developed the least severe colitis; feeding egg white protein, casein protein, or soy protein diets resulted in intermediate colitis severity (Figure 1*A–D*). Similarly, PP feeding resulted in less severe colitis compared with other dietary protein sources, including BP, in a T-cell–mediated model using sex-balanced, ex germ-free (GF) *Il10*^−/−^ mice colonized with mouse-adapted pooled human IBD microbiota (IMM-HM2)^32^ (Figure 1*E–H*). This was characterized by less histologic inflammation, lower fecal lipocalin-2 levels, and lower colonic proinflammatory cytokine expression in PP-fed vs BP-fed mice (Figure 1*E–H*). These differences in colitis severity were sex-independent (https://github.com/simonmgray/dietary_protein_source_mediates_experimental_colitis).

**Table 1:** Experimental Diet Formulations

Ingredient	CP	SP	EP	PP	BP	BP + psyllium
	*g*	*g*	*g*	*g*	*g*	*g*
Casein	200	0	0	0	0	0
SP, Supro 661	0	202.3	0	0	0	0
Egg whites	0	0	210.9	0	0	0
BP Isolate, Sigma	0	0	0	0	226.9	226.9
PP, Pure Bulk	0	0	0	209.1	0	0
L-cystine	3	3	3	3	3	3
Corn starch	397.486	395.486	389.286	398.486	398.486	385.329
Maltodextrin 10	132	132	132	132	132	132
Sucrose	100	100	100	100	100	100
Psyllium	0	0	0	0	0	52.63
Cellulose, BW200	50	50	50	50	50	0
Soybean oil	70	63	70.2	51.8	70.8	70.8
t-Butylhydroquinone	0.014	0.014	0.014	0.014	0.014	0.014
Mineral mix S10022G	0	0	0	0	0	0
Mineral mix S10001A	17.5	17.5	17.5	17.5	17.5	17.5
Calcium phosphate, dibasic	17.5	17.5	17.5	17.5	17.5	17.5
Calcium carbonate	0	0	0	0	0	0
Potassium citrate, monohydrate	0	0	0	0	0	0
Potassium phosphate, monobasic	0	0	0	0	0	0
Sodium chloride	20.2	19.2	12.9	19.3	0	0
Vitamin mix V10037	0	0	0	0	0	0
Vitamin mix V10001	10	10	10	10	10	10
Biotin, 0.1%	0	0	0.4	0	0	0
Choline bitartrate	2.5	2.5	2.5	2.5	2.5	2.5
Total	1020.2	1012.5	1016.2	1011.2	1028.7	1018.2
Protein, *g*	177.0	177.0	177.0	177.0	177.0	177.0
Carbohydrate, *g*	640.5	640.5	640.5	640.5	640.5	640.5
Fat, *g*	71.0	71.0	71.0	71.0	71.0	71.0
Fiber, *g*	50.0	50.0	50.0	50.0	50.0	50.0
Protein, *kcal*	708.0	707.9	708.0	707.9	708.1	708.1
Carbohydrate, *kcal*	2561.9	2561.8	2561.9	2561.9	2561.9	2561.9
Fat, *kcal*	638.6	639.3	639.4	639.3	639.2	639.2
Total	3908.6	3909.0	3909.3	3909.2	3909.3	3909.3
Protein, *g %*	17.3	17.5	17.4	17.5	17.2	17.2
Carbohydrate, *g %*	62.8	63.3	63.0	63.3	62.3	63.0
Fat, *g %*	7.0	7.0	7.0	7.0	6.9	6.9
Protein, *kcal %*	18.1	18.1	18.1	18.1	18.1	18.1
Carbohydrate, *kcal %*	65.5	65.5	65.5	65.5	65.5	65.5
Fat, *kcal %*	16.3	16.4	16.4	16.4	16.4	16.4
Sodium, *g/kg*	8.8	8.8	8.8	8.8	8.8	8.8
*kcal/g*	3.8	3.9	3.8	3.9	3.8	3.8

BP, beef protein; CP, casein protein; EP, egg white protein; PP, pea protein; SP, soy protein.

**Figure 2:**
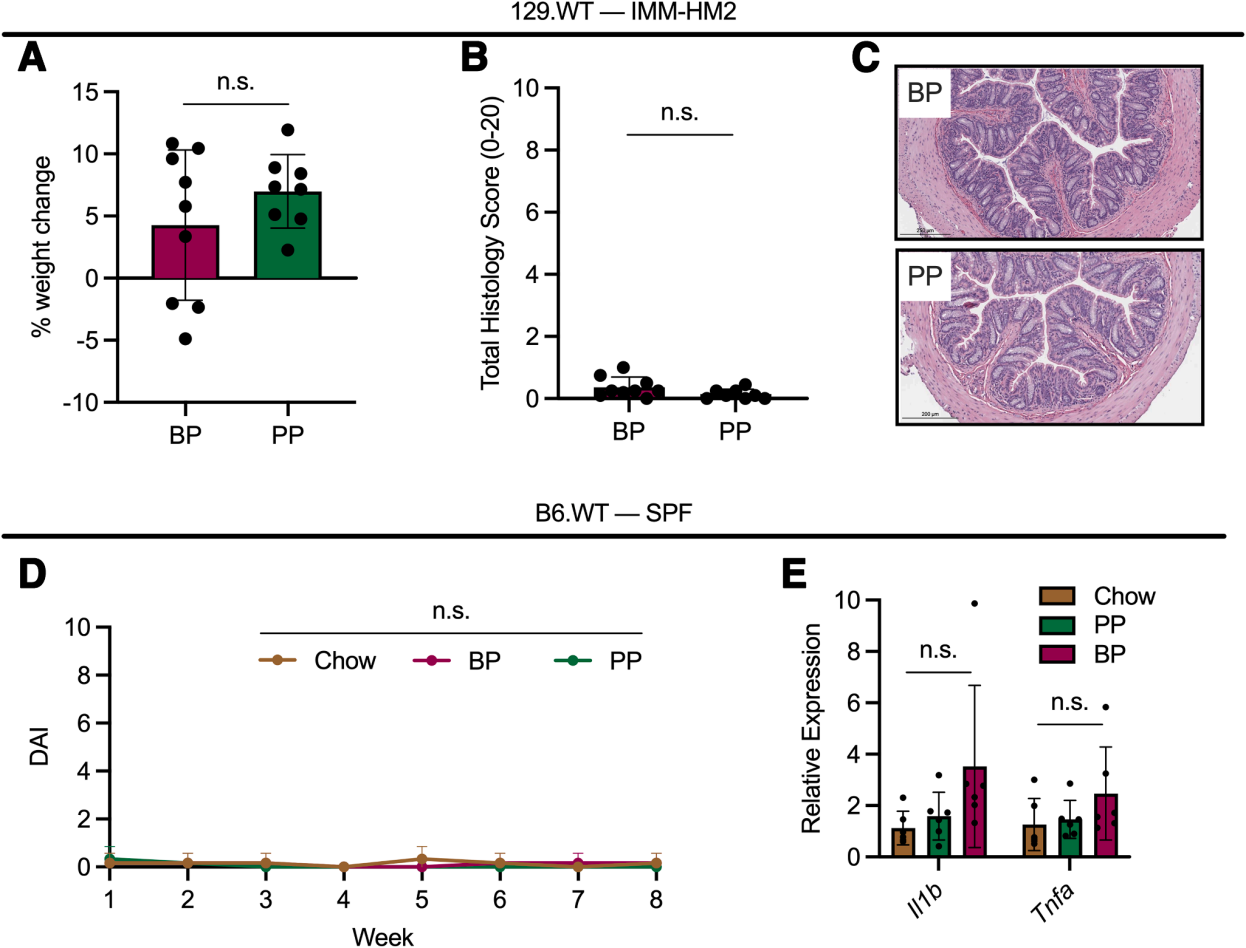
**BP feeding does not induce colonic inflammation in the absence of a colitis inducer.** (*A–C*) GF 129S6/SvEv WT mice were inoculated with MA pooled human IBD patient fecal microbiota (IMM-HM2) then fed BP or PP diets for 21 days. Weight changes relative to day 0 were measured (*A*), histology score was assessed (*B*), and representative histologic images of colons are shown (*C*), on day 14. n = 8–9 mice/group. (*D* and *E*) C57BL/6J WT SPF mice were fed chow, or purified diet containing BP or PP isolate for 8 weeks. The DAI was measured weekly (*D*), and relative expression of *Tnfa* and *Il1b* was measured in colons on week 8 (*E*). n = 6 mice per group. Bar plots show mean and standard deviation.

To evaluate whether BP consumption alone promotes colonic inflammation in the absence of a colitis inducer (ie, *Il10* deficiency or DSS) WT mice were colonized with IMM-HM2 microbiota and fed a BP or PP diet for 21 days, which showed no overt colitis in either diet group (Figure 2*A–C*). Long-term feeding of a PP or BP diet or standard chow for 8 weeks in WT SPF mice similarly showed no significant differences in colitis parameters including Disease Activity Index (DAI) or colonic proinflammatory cytokine expression (Figure 2*D* and *E*). Collectively, these data demonstrate that the protein component of beef worsens colonic inflammation in multiple experimental colitis models specifically in the presence of a colitis trigger.

### Bacteria Drive Colitis Severity in Beef Protein–Fed Mice

**Figure 3:**
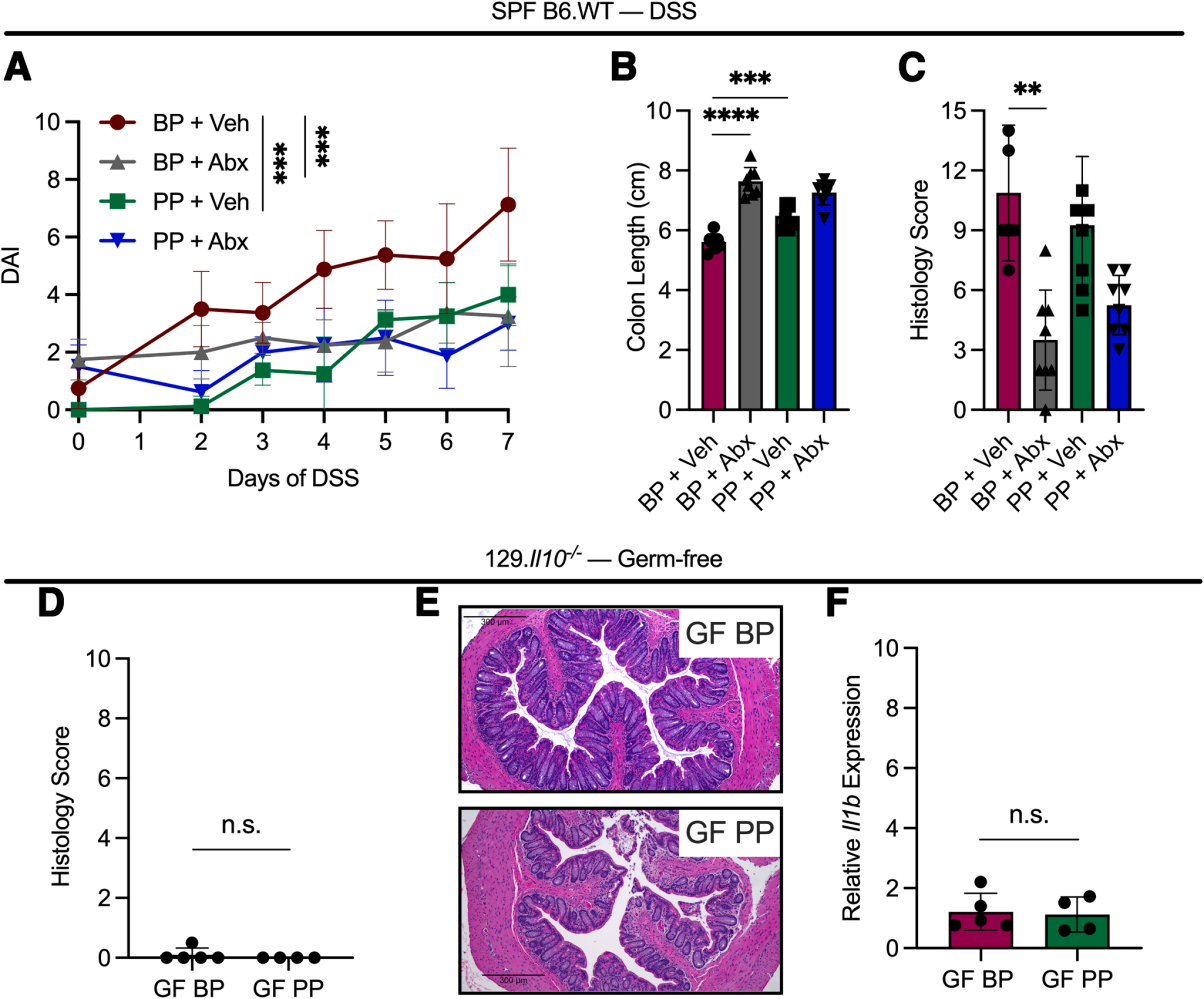
**Gut bacteria are required for BP-worsening of colitis.** (*A–C*) C57BL/6J mice were fed BP- or PP-containing diets and given regular drinking water (Veh) or fed either diet and administered a 5-antibiotic cocktail (ampicillin, gentamicin, metronidazole, neomycin, vancomycin^21^) in drinking water for 14 days (Abx), then all mice were given 1% DSS in drinking water for 7 days while being continued on Veh or Abx. The DAI was measured during DSS exposure (*A*), and colon length (*B*) and histologic inflammation (*C*) were assessed on day 7. n = 8 mice per group. (*D–F*) GF 129S6/SvEv *Il10*^*−/−*^ mice were fed sterile BP or PP diets for 14 days. Histology score was assessed in colons (*D*), representative histologic images of colons are shown (*E*), and relative gene expression of *Il1b* was measured in colons (*F*), on day 14. n = 4–5 mice per group. ∗∗*P* < .01; ∗∗∗*P* < .001; ∗∗∗∗*P* < .0001. Significance indicators for (*A*) represent differences in values over 7 days. Bar plots show mean and standard deviation.

**Figure 4:**
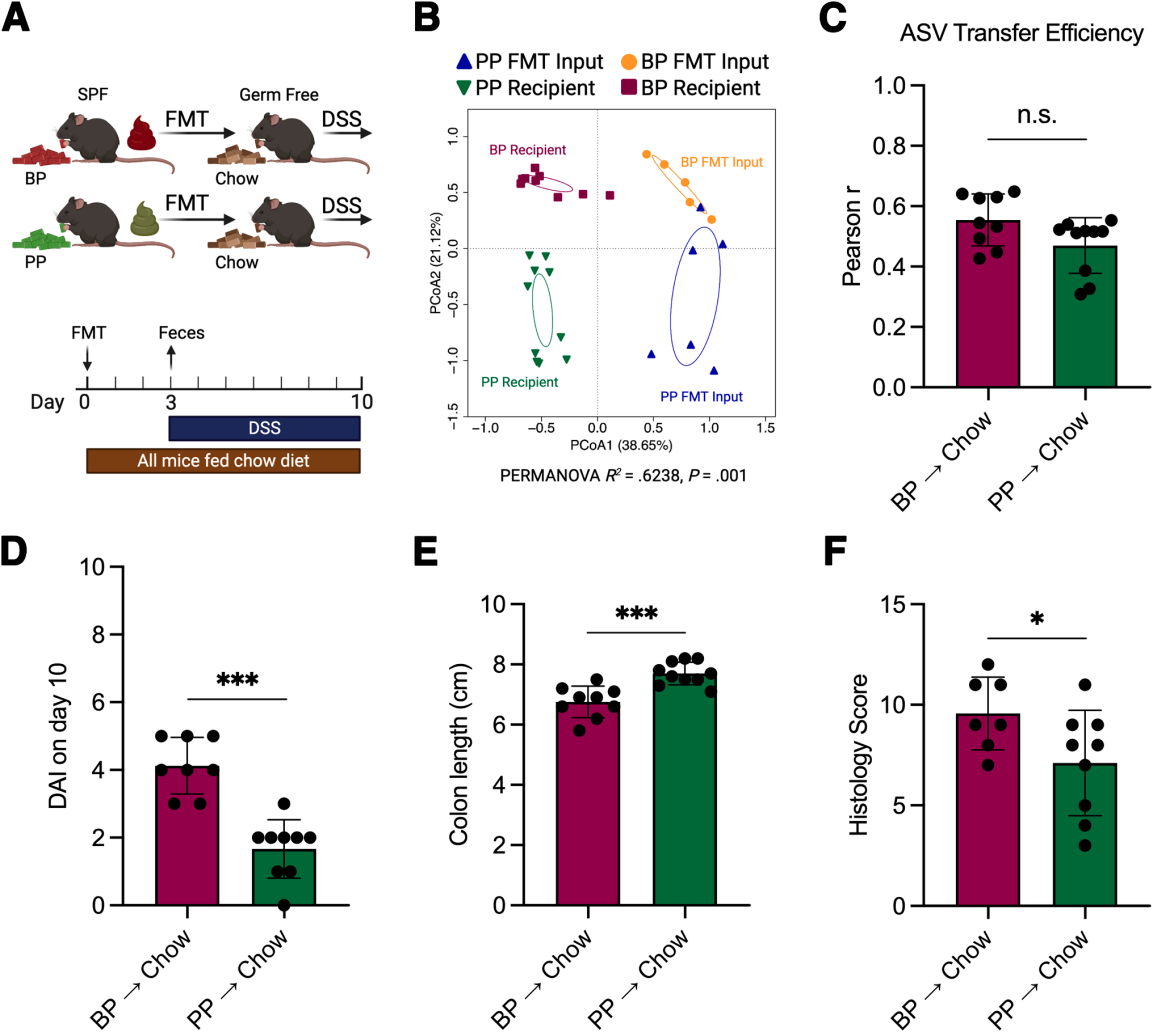
**Gut bacteria are causally linked to BP-worsening of colitis.** (*A–F*) GF C57BL/6J WT mice were inoculated with colonic luminal fecal contents of SPF mice fed BP or PP diets for 7 days. Recipient mice were then fed standard chow and administered 1% DSS for 7 days, starting 3 days after colonization (*A*). Donor FMT samples and feces of recipient mice 3 days after colonization were subjected to 16S rRNA sequencing, PCoA of donor FMT samples and recipient mice was plotted (*B*), and amplicon sequence variant (ASV) transfer efficiency was quantified by Pearson r of donor-recipient pairs (*C*). DAI (*D*), colon length (*E*), and histology score (*F*) were determined on day 10 (7 days after initiating DSS). n = 9–10 per group; data are representative of 2 independent experiments. ∗*P* < .05; ∗∗∗*P* < .001. Bar plots show mean and standard deviation. PERMANOVA, permutational multivariate analysis of variance.

We next tested whether protein source modulates colitis severity, in part, through gut resident bacteria. First, WT SPF mice were fed a PP diet or BP diet and given plain drinking water or water containing broad-spectrum antibiotics to globally reduce gut bacteria, then challenged with DSS while continuing the respective diets and drinking water. Antibiotic treatment reduced BP diet–mediated DSS colitis severity to, or below, PP diet–mediated levels (Figure 3*A–C*). Bacterial depletion in antibiotic-treated mice was confirmed by performing quantitative real-time polymerase chain reaction (qRT-PCR) on DNA extracted from feces, demonstrating a 98.8% decrease in total bacterial DNA compared with vehicle-treated mice (0.01 ± 0.02 vs 1.03 ± 0.23 for relative bacterial abundance; *P* = .0008). Next, GF *Il10*^−/−^ mice were fed sterile BP or PP diets for 14 days and assessed for colonic inflammation by multiple parameters, which revealed no overt colitis in either group (Figure 3*D–F*). GF status in both groups was confirmed by aerobic and anaerobic culture of feces, with no growth observed in GF samples. Lastly, we performed a fecal microbial transplant phenotype transfer experiment. Here, feces from WT SPF mice fed BP or PP diets for 1 week were transplanted to GF WT mice fed standard rodent chow and challenged with DSS for 7 days (Figure 4*A*). Transfer efficiency measurements using donor fecal microbiota transplantation (FMT) material and recipient mice feces 3 days after FMT (but before DSS administration) revealed similar clustering of donor samples and matched FMT recipients along Principal Coordinate Axis 2 (PCoA2), whereas donors and recipients clustered separately along Principal Coordinate Axis 1 (PCoA1) regardless of dietary protein group (Figure 4*B*). Moreover, comparisons of amplicon sequence variant abundances between donor FMT and matched recipient mice samples demonstrated a high Pearson r, confirming high and equally efficient transfer efficiency in both groups (Figure 4*C*).^32^ Measurements of DAI, colon length, and histologic score showed that mice gavaged with fecal slurry from BP-fed mice developed more severe colitis than mice administered feces from PP-fed mice (Figure 4*D–F*). Together, these data demonstrate that BP exacerbates colitis through the diet-altered microbiota.

### Beef Protein Feeding Expands *A muciniphila* and Impairs the Gut Barrier

**Figure 5:**
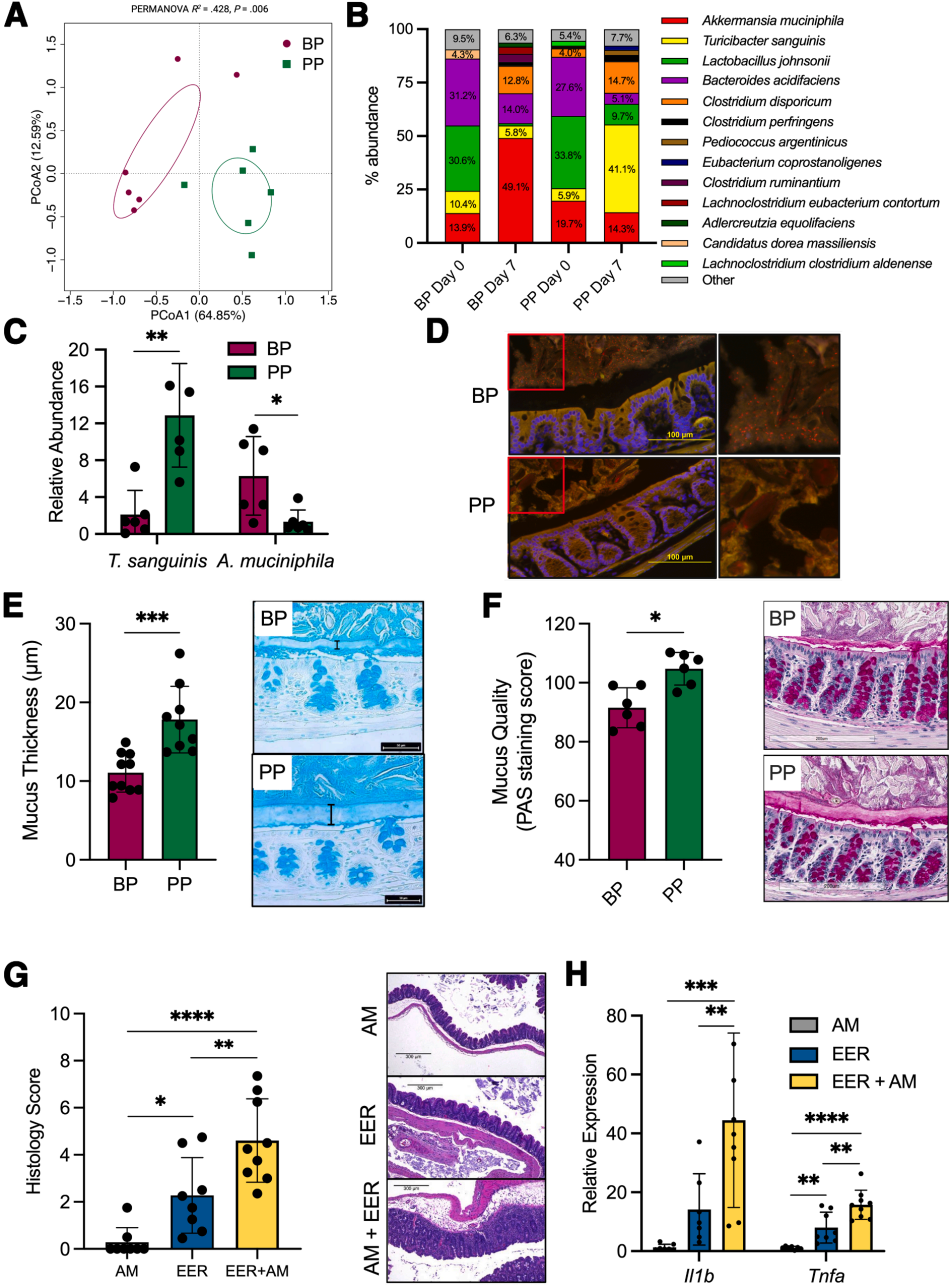
**BP consumption promotes *Akkermansia muciniphila* expansion and gut barrier defects.** (*A–C*) C57BL/6J mice were fed either BP- or PP-containing diets for 7 days, then fecal samples were subjected to 16S rRNA sequencing. Results are displayed as PCoA on day 7 (*A*) and as percent abundance of each species on days 0 and 7 (*B*) (those species <1% in overall abundance are labeled as ‘Other’). qRT-PCR was performed on fecal DNA to measure relative abundance of major populations found in either diet group (*C*). (*D–F*) C57BL/6J WT mice were fed BP or PP diets for 7 days, then colons were harvested and fixed to preserve the mucus layer. FISH was performed to identify mucus-associated *A muciniphila* (*Cy-3 labeled orange structures*) and 4′,6-diamidino-2-phenylindole (DAPI) was used to stain nuclei of colon mucosa cells (*D*). Alcian blue staining was carried out, and mucus thickness was measured (quantification of staining shown on *left side* and representative images shown on *right side*; bracket indicates mucus layer on images) (*E*), and Periodic acid Schiff (PAS) staining was performed, and mucus quality was quantified (quantification of staining shown on *left side* and representative images shown on *right side*) (*F*). n = 6 per group. (*G* and *H*) GF 129S6/SvEv *Il10*^*−/−*^ mice were inoculated with *A muciniphila* (AM), a defined consortium of *Escherichia coli*, *Enterococcus faecalis*, and *Ruminococcus gnavus* alone (EER) or in combination with *A muciniphila* (EER+AM) and fed BP diet. Total histology score was assessed in ceca (*left side*), and representative histologic images of ceca are shown (*right side*) (*G*), and relative expression of inflammatory cytokine genes was measured in the ceca of mice (*H*), on day 14. n = 7–9 per group; data are representative of 2 independent experiments. ∗*P* < .05; ∗∗*P* < .01; ∗∗∗*P* < .001; ∗∗∗∗*P* < .0001. Bar plots show mean and standard deviation.

To characterize the dietary protein–driven microbiota that mediate colitis severity, bacterial community composition was profiled by 16S sequencing (16S-seq) of feces from SPF WT mice fed BP or PP diets for 1 week in the absence of colitis. PCoA demonstrated distinct clustering of mice according to dietary protein source, which explained approximately 65% of variation in the data in PCoA1 (permutational multivariate analysis of variance; *R*^*2*^ = .43; *P* = .005) (Figure 5*A*). Although profiles before feeding either experimental diet were similar, 7 days of feeding a BP or PP diet significantly altered the taxonomic abundance of multiple bacteria (Figure 5*B*). Significant expansion of *A muciniphila* occurred in the BP diet group, whereas *Turicibacter sanguinis* increased in the PP diet group, along with partial preservation of *Lactobacillus johnsonii* (Figure 5*B* and *C*). *A muciniphila* can impair gut barrier function through mucus digestion^16^^,^^21^^,^^25^^,^^33^, ^34^, ^35^; therefore, we examined its colonic luminal spatial distribution by fluorescence in situ hybridization (FISH). This revealed high abundance of *A muciniphila* adjacent to the mucus layer of BP-fed mice, whereas it was absent in PP-fed mice (Figure 5*D*). Colonic surface mucus thickness showed a corresponding ∼50% reduction in BP vs PP fed mice (Figure 5*E*). Additionally, surface mucus quality was reduced in BP- vs PP-fed mice (Figure 5*F*). To evaluate whether BP-driven expansion of *A muciniphila* was responsible, in part, for promoting colitis, GF *Il10*^−/−^ mice were selectively colonized with *A muciniphila* alone, a consortium of IBD-associated pathobionts including *Escherichia coli*, *Enterococcus faecalis*, and *Ruminococcus gnavus* (EER), or *A muciniphila* plus EER and fed a BP diet. Although *A muciniphila* mono-association did not induce colitis, it significantly potentiated EER pathobiont–induced colitis severity (Figure 5*G* and *H*). Appropriate strain colonization was confirmed by anaerobic plating of feces from colonized mice followed by 16S colony PCR and Sanger sequencing of representative colony morphologies. These data suggest that BP exacerbates colitis, in part, by promoting expansion and mucus layer localization of mucus-digesting *A muciniphila* to potentiate the proinflammatory effects of IBD-associated pathobionts.

### High Levels of Conjugated Bile Acids Promote Colitis Severity During Beef Protein Feeding

**Figure 6:**
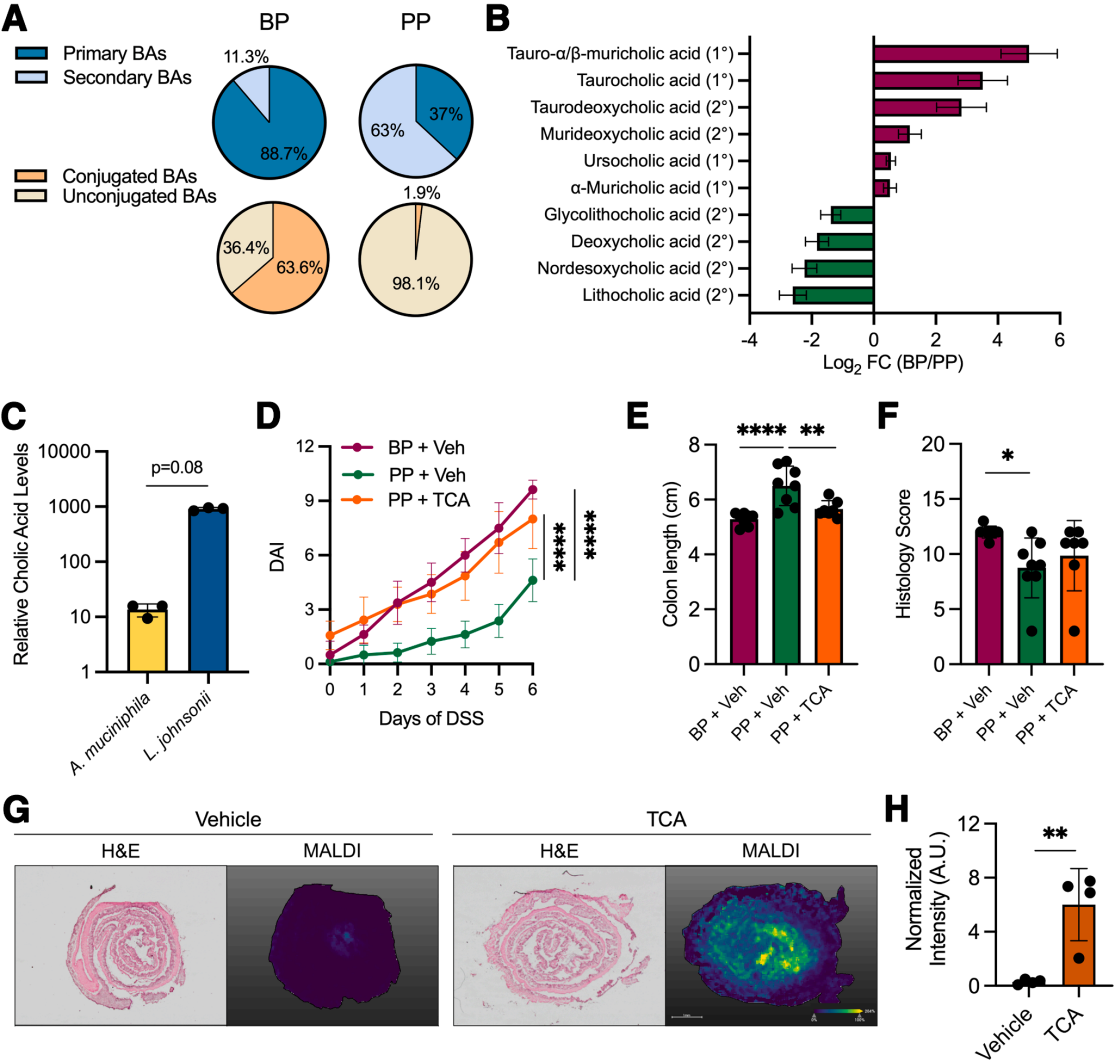
**Diet-induced BA changes mediate colitis severity.** (*A* and *B*) C57BL/6J WT mice were fed either BP- or PP-containing diets for 7 days and the relative abundance of BAs was measured in feces. The average percent of primary vs secondary or CBAs vs unconjugated BAs in each diet group is shown (*A*) and those BAs that displayed significantly different abundance (false discovery rate [FDR] <0.1) are shown as log2 fold-change (FC) in PP-fed mice relative to BP-fed mice (*B*). (C) TCA (1 mM) was administered to culture medium of *Lactobacillus johnsonii* and *Akkermansia muciniphila* and the levels of cholic acid were measured in the supernatants after 24 hours and reported as relative to either species cultured in the absence of TCA. (*D–F*) C57BL/6J WT mice were fed either BP or PP diets and given daily oral gavage of PBS or fed PP and gavaged daily with TCA (0.35 mg/kg) for 7 days, then administered 1% DSS in drinking water for 7 days, while continuing oral administration of PBS or TCA. DAI was measured over 7 days (*D*), colon length was measured on day 7 (*E*), and histologic inflammation of colons was assessed on day 7 (*F*). (*G* and *H*) C57BL/6J WT mice were administered daily oral gavage of PBS or TCA (0.35 mg/kg) for 7 days, and colon tissue sections were subjected to MALDI-MS imaging to determine relative abundance of TCA in each group. Representative images of H&E-stained colon tissue sections and corresponding TCA signal are shown in serial sections (*G*) and abundance of TCA in colons from the TCA treated group was quantified relative to the vehicle-treated group (*H*). n = 4 samples per group. ∗*P* < .05; ∗∗*P* < .01; ∗∗∗∗*P* < .0001. Bar plots show mean and standard deviation.

Because bacteria have an important role in mediating BA metabolism and composition and because there was a higher abundance of bile salt hydrolase–carrying bacteria (*T sanguinis*, *L johnsonii*)^36^, ^37^, ^38^, ^39^, ^40^ in PP-fed mice, we next measured the relative levels of common BAs in feces from WT SPF mice fed BP or PP diets for 7 days in the absence of colitis. This analysis showed that most BAs in BP-fed mice were primary and conjugated, whereas most BAs in PP-fed mice were secondary and unconjugated (Figure 6*A*). Multiple taurine-conjugated primary BAs, including taurocholic acid (TCA), were significantly higher in BP-fed mice whereas several unconjugated secondary BAs, including lithocholic acid and deoxycholic acid, were elevated in PP-fed mice (Figure 6*B*). These patterns were not observed in the livers or ileal content of mice fed BP vs PP diets, suggesting that fecal BA alterations are likely mediated by colon-specific microbial differences. To connect the respective BA profiles of mice fed PP or BP diets with differential bacterial abundance, we performed an in vitro BA deconjugation assay comparing select bacterial species enriched by either diet. This revealed that *L johnsonii* (higher abundance in PP-fed mice) deconjugated TCA to cholic acid 100-fold more than *A muciniphila* (higher abundance in BP-fed mice) (Figure 6*C*). To evaluate whether the primary CBAs that were increased in feces of BP diet–fed mice directly exacerbate colitis, mice given PP were administered TCA by oral gavage daily for 7 days and exposed to DSS. Measurements of multiple parameters of colitis severity revealed that TCA administration worsened colitis in PP-fed mice (Figure 6*D–F*). Subsequent matrix-assisted laser desorption/ionization (MALDI)-MS imaging–based visualization of the colons of mice administered TCA in the absence of colitis revealed TCA accumulation in the colonic epithelium (Figure 6*G* and *H*). Collectively, these data suggest that mice consuming a BP-containing diet have lower abundance of BA-deconjugating bacteria, resulting in higher levels of colitis-promoting primary CBAs.

### Inclusion of Psyllium in a Beef Protein Diet Reduces Colitis Severity and Promotes Bile Acid Deconjugation

**Figure 7:**
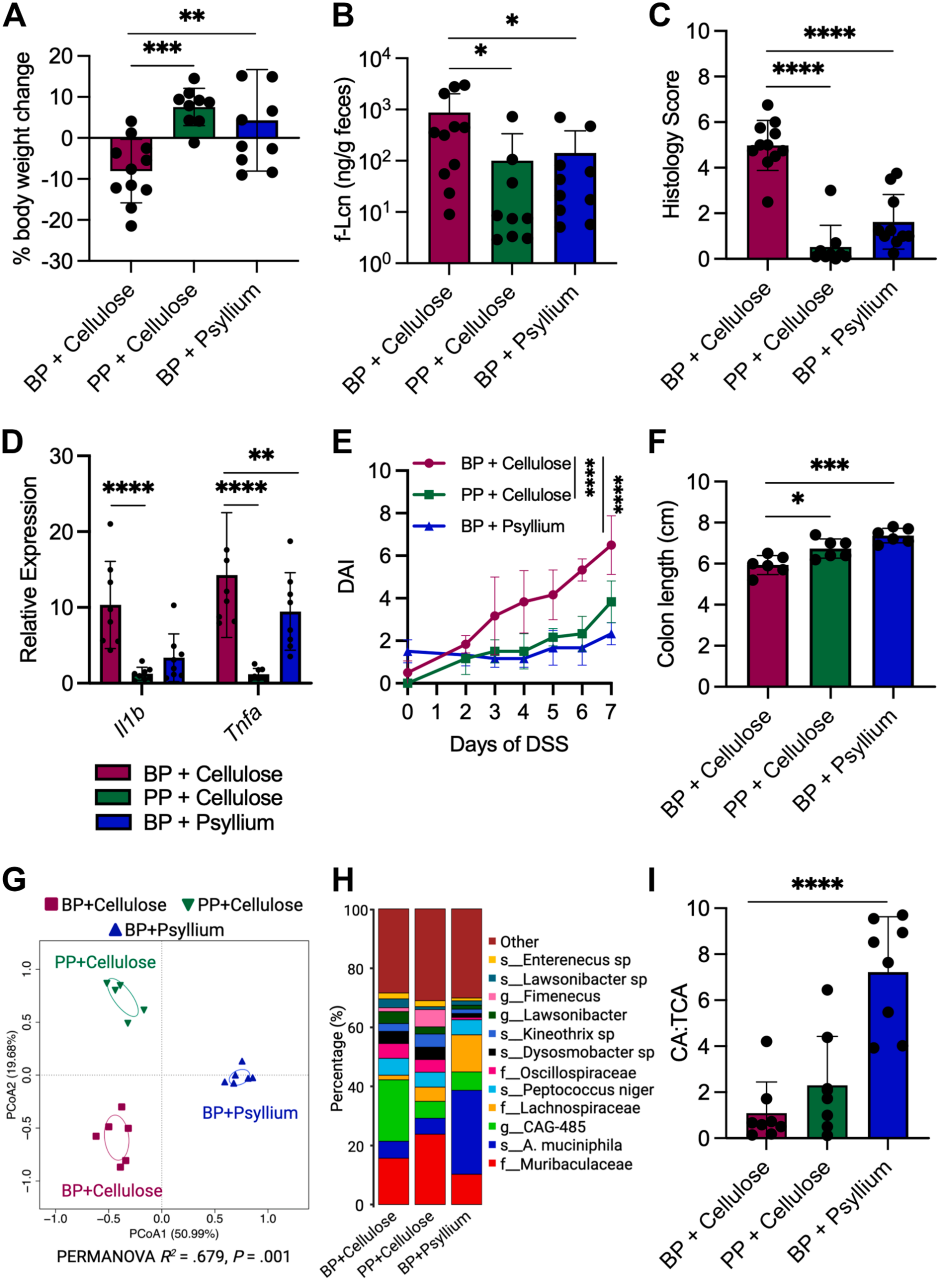
**Psyllium supplementation protects against dietary BP-induced worsening of colitis and normalizes BA ratios.** (*A–D*) GF 129S6/SvEv *Il10*^*−/−*^ mice were inoculated with MA pooled human IBD patient fecal microbiota (IMM-HM2) then fed BP (BP + Cellulose) or PP (PP + Cellulose) diets containing cellulose or BP diet containing psyllium (BP + Psyllium) for 14 days. Weight changes relative to day 0 were calculated (*A*), fecal lipocalin-2 (f-Lcn) was measured (*B*), total histology score was assessed (*C*), and relative gene expression of inflammatory cytokines in colons was measured (*D*), on day 14. n = 10–11 mice per group. (*E* and *F*) C57BL/6J WT SPF mice were fed the diets described in (*A*) for 7 days. Mice were then administered 1% DSS, and the DAI was measured over 7 days (*E*), and colon length was measured on day 7 (*F*). (*G* and *H*) C57BL/6J WT SPF mice were fed the diets described in (*A*) for 7 days, and feces were collected and subjected to 16S rRNA sequencing. Data are shown as PCoA (*G*) and taxonomic bar plots showing the top 12 most abundant taxa (*H*). (*I*) Fecal samples described in (*G*) were used to measure the ratio of cholic acid (CA) to TCA. n = 10–11 mice per group. ∗*P* < .05; ∗∗*P* < .01; ∗∗∗*P* < .001; ∗∗∗∗*P* < .0001. Significance indicators for (*E*) represent differences in values over 7 days. Bar plots show mean and standard deviation.

Microbe-accessible dietary fiber supports populations of gut health–promoting commensals, including BA-metabolizing bacteria.^41^ Because our diets were formulated with non-microbe–accessible cellulose fiber, we reasoned that adding soluble dietary fiber to BP diet would maintain gut health–promoting bacterial populations and attenuate BP-driven colitis severity. To test this concept, ex-GF *Il10*^−/−^ mice inoculated with mouse-adapted (MA) human IBD microbiota were fed a PP-cellulose diet, a BP-cellulose diet, or a BP diet in which cellulose fiber was replaced with psyllium (BP-psyllium diet) and monitored for colitis severity (Table 1). Compared with the BP-cellulose diet, the BP-psyllium diet significantly decreased colitis severity, as determined by body weight measurements, fecal lipocalin levels, histologic analysis, and inflammatory cytokine gene expression (Figure 7*A–D*). Similar protection by psyllium was observed in the DSS-induced colitis model (Figure 7*E* and *F*). 16S-seq was then performed to investigate the microbiome composition driven by psyllium feeding in the absence of colitis. PCoA demonstrated distinct clustering of each of the 3 diets (permutational multivariate analysis of variance; *R*^*2*^ = .679, *P* = .001) (Figure 7*G*). Specifically, groups clustered distinctly by dietary fiber type (cellulose vs psyllium) along the PCoA1, which explained 50.99% of variation in the data, whereas the BP-cellulose and PP-cellulose groups clustered separately along PCoA2 (Figure 7*G*). Taxonomic analysis demonstrated that the BP-psyllium diet induced a compositionally distinct microbiome compared with the BP-cellulose and the PP-cellulose diets (Figure 7*H*). Notably, this bacterial profile was associated with a higher cholic acid:TCA ratio compared with BP-cellulose feeding, suggesting a rebalancing of CBAs to unconjugated BAs (Figure 7*I*). Taken together, these data support a model in which limited conversion of CBAs to their deconjugated form, driven by a BP diet, can be overcome by restoring populations of BA-modulating bacteria through psyllium fiber administration, thereby attenuating diet-driven exacerbation of colitis.

## Discussion

Plant-based diets are believed to benefit patients with IBD, whereas animal-based diets, particularly red meat, may be detrimental. Both retrospective and prospective studies suggest that consumption of red meat is associated with IBD incidence and disease activity. For example, The E3N Prospective Cohort Study revealed that high animal protein consumption significantly increased IBD incidence,^13^ and multiple studies showed that meat intake increased the risk of disease flare in patients with ulcerative colitis.^5^^,^^12^^,^^14^^,^^15^ The concept that plant-based diets are beneficial largely stems from studies demonstrating that microbe-fermentable carbohydrates from plants reduce experimental colitis.^2^^,^^42^ In contrast, diets rich in animal-derived products (especially red meat) are thought to promote intestinal inflammation through heme, sulfur, and saturated fat content.^2^^,^^42^ Although these components of plant- and animal-based diets impact gut health, the role of protein content has not been widely considered. Our study, which selectively examined protein content by feeding mice diets that differed only in the type of protein isolate they contained, suggests that the source of dietary protein content differentially impacts colitis severity. Other studies investigating the impact of protein source on experimental colitis, including from beef and pea, are largely consistent with the data shown in the current study, although this previous work used diets containing whole food sources rather than purified protein isolate.^18^^,^^19^^,^^43^^,^^44^ Our findings support a role for the dietary protein-altered microbiota and BAs in mediating colitis severity; however, the specific mechanism(s) by which protein type drives these effects remains unclear. Dietary protein sources vary in trace minerals, such as iron, which has been shown to potentiate experimental colitis.^45^ However, the BP isolate used in this study had lower iron content than the PP isolate (0.0015% vs 0.0248%), making this explanation unlikely (Table 2). It is plausible that differences in amino acid composition or digestion/absorption of diverse dietary protein sources are contributing factors. Therefore, determining which amino acids or what properties of each protein type mediate intestinal inflammation will be important for future studies.

**Table 2:** Trace Mineral Analysis of Pure Protein Isolates

Trace mineral	BP isolate	PP isolate
Copper (Cu)	1.5 ppm	11 ppm
Iron (Fe)	0.0015%	0.0248%
Magnesium (Mg)	0.058%	0.074%
Manganese (Mn)	2.4 ppm	16 ppm
Zinc (Zn)	41 ppm	109 ppm

BP, beef protein; PP, pea protein.

Bacteria, BAs, and their interplay are key mediators of IBD pathogenesis.^1^^,^^2^ For example, higher levels of CBAs and primary BAs are found in feces of patients with active IBD and dysbiosis, compared with greater amounts of unconjugated BAs in healthy individuals.^27^, ^28^, ^29^ Our work suggests that a shift to a predominance of CBAs and primary BAs is, in part, responsible for greater colitis severity when feeding BP to mice. Although the exact mechanism(s) by which altered BAs enhance disease is unclear, the detergent-like properties of BAs could disrupt gut barrier function by impairing epithelial cell function and/or epithelial-protective mucus. This concept is supported by our data showing the ability of exogenously administered TCA to localize to the colonic epithelium. Interestingly, our prior work showed that a high-fructose diet exacerbated murine colitis with a similar increase in CBAs as observed in BP-fed mice, with rectal administration of CBAs worsening colitis and thinning colonic mucus in mice fed a control diet.^21^ Integrating our study focused on dietary protein with prior studies of the role of dietary fat and carbohydrates in experimental colitis suggests that altered BAs may be a common pathway for diet-mediated gut inflammation.^21^^,^^26^ Although our work focused on the colitis-promoting effects of conjugated primary BAs enriched in BP-fed mice, the profile of unconjugated, secondary BAs enriched in feces from PP-fed mice might itself be protective. For example, lithocholic acid and deoxycholic acid, both enriched in PP-fed mice, are strong ligands of the pregnane X receptor and Takeda G protein–coupled receptor 5 that, when activated, induce anti-inflammatory effects.^28^^,^^46^, ^47^, ^48^ Therefore, the role of unconjugated secondary BAs as protective metabolites in PP-fed mice is an important future direction.

BAs have a bidirectional relationship with gut bacteria, whereby bacterial enzymes metabolize BAs, which in turn, alter bacterial function and microbiota composition.^46^^,^^49^ PP feeding in the current work expanded bacterial species that efficiently deconjugate BAs, including *L johnsonii* and *T sanguinis*, as shown by the results of the deconjugation assay in this study and previous work.^21^^,^^37^^,^^50^ In contrast, BP feeding increased abundance of *A muciniphila,* a poor deconjugator that also digests mucus and potentiates pathobiont-driven inflammation.^51^ Although dietary protein–induced bacterial shifts likely caused the observed BA profiles, we cannot exclude the possibility that altered BAs directly shifted microbiota composition, as previously demonstrated.^52^, ^53^, ^54^, ^55^ Further evidence that the ratio of CBAs to unconjugated BAs is important for mediating colitis stems from our findings that psyllium supplementation protected against BP-mediated colitis severity, while maintaining favorable BA profiles and populations of BA-deconjugating bacteria. However, psyllium fiber likely protects against inflammation by multiple mechanisms, including short-chain fatty acid production and prevention of bacterial mucus degradation.^16^^,^^56^

The beneficial or deleterious impact of certain gut bacteria is increasingly recognized to be context-dependent.^57^ In IBD, there are conflicting data showing *A muciniphila* capable of both promoting and attenuating gut inflammation, with strain-level genetic variation proposed as a critical factor.^57^ We found that, in the absence of microbially accessible dietary fiber (BP-cellulose diet), *A muciniphila* potentiated pathobiont-induced colitis in a defined consortium study of selectively colonized *Il10*^*−/−*^ mice. However, in the presence of psyllium (BP-psyllium diet), a microbially accessible fiber, *A muciniphila* increased in relative abundance, whereas gut inflammation was ameliorated. Therefore, our results affirm the emerging evidence^51^ that *A muciniphila* acts in a dietary context–dependent manner to be beneficial or deleterious to the host.

Our study supports the concept that IBD occur when multiple detrimental factors intersect to initiate and perpetuate gut inflammation.^58^ These IBD determinants—genetically dysregulated host inflammatory and epithelial barrier responses, abnormal resident microbes, and environmental triggers—can exist in isolation or single combination without causing disease, but together drive severe IBD. For example, GF colitis-susceptible *Il10*^*−/−*^ mice fed a BP diet (genetic susceptibility + environmental trigger) did not develop inflammation, nor did human IBD microbiota-colonized WT mice fed a BP diet (abnormal resident microbes + environmental trigger). However, we demonstrated that when all 3 IBD-driving factors—host susceptibility, microbe, and environment—intersect, severe and progressive intestinal inflammation occurs. The poor long-term success of current human IBD therapies may reflect a failure to reconcile the multiple determinants of IBD. For example, addressing just 1 factor, such as suppression of host inflammation, leaves the patient vulnerable to recurrent disease from persistent dysbiosis and upon encountering environmental triggers.^59^ We suggest that durable long-term remission requires simultaneously addressing all IBD determinants, and as such, investigations into environmental drivers of IBD, especially diet, are required to guide clinical approaches to maintain sustained remission.

## Materials and Methods

All authors had access to the study data and reviewed and approved the final manuscript.

### Sex as a Biological Variable

Our study examined male and female animals, and similar findings are reported for both sexes. Linear mixed-effects modeling was used to test for sex effects as outlined in the statistical analysis section.

### Murine Colitis Models

#### Randomization

(1) In SPF mouse studies, mice were equally randomized to groups after receipt from vendors. Bedding swapping was performed for 2 weeks between cages to homogenize microbiota and minimize cage effects before study initiation. (2) In ex-GF mouse studies, littermates were randomized equally to groups prior to colonization. When mice from multiple breeder isolators were used for 1 study, mice were equally randomized by litter and breeder isolator origin prior to colonization. All groups had equal numbers of male and female mice.

To induce chemical colitis, 8-week-old male SPF C57BL/6J mice (The Jackson Laboratory) were administered 1% DSS (Sigma) in drinking water for 6 to 7 days, as indicated. Colitis severity was assessed by measuring changes in body weight, as well as severity of rectal bleeding and diarrhea, as previously described.^21^^,^^60^ The DAI was calculated by combining the severity of body weight loss, diarrhea, and bleeding, as previously described.^21^^,^^60^ Purified isocaloric diets were generated by Research Diets, Inc, containing protein isolate from beef (Sigma), soy (Solae), casein (Fonterra), egg whites (Debel), or pea (Purebulk, Inc) and fed to mice for 1 week prior to DSS administration (Table 1).

T-cell–mediated colitis was induced by inoculation of mouse-adapted human IBD patient fecal microbiota (previously developed and characterized^32^) to 8- to 12-week-old, GF male and female *Il10*^*−/−*^ mice or WT (noninflamed control) on a 129S6/SvEv background (purchased from the National Gnotobiotic Rodent Resource Center [NGRRC] at the University of North Carolina), as previously described.^32^ MA human IBD patient microbiota was previously generated from deidentified human IBD patient stool samples collected under an Institutional Review Board–approved protocol; no primary human samples were used in this study.^32^ Briefly, human fecal materials from pooled cohorts of human donors with active IBD were passaged through GF 129S6/SvEv *Il10*^*−/−*^ mice to generate standardized aliquots of fecal slurry.^32^ MA IMM-g2 microbiota was derived from pooled feces of 2 patients with Crohn’s disease and 1 patient with ulcerative colitis, and MA IMM-HM2 microbiota was derived from pooled feces of 3 patients with Crohn’s disease.^32^ Standardized 100 mg/mL aliquots of MA human IBD microbiota were thawed under anaerobic conditions, diluted with prereduced phosphate-buffered saline (PBS), and administered by oral gavage (2 mg) to recipient GF *Il10*^*−/−*^ mice.^32^ All fecal transplant experiments were performed with sterile gnotobiotic cage technique in BSL-2 isolation cubicles with HEPA-filtered air.^61^ Prior to colonization, GF mice were fed Purina Advanced Protocol Select Rodent 50 IF/6F Auto Diet. Defined diets were started at the time of FMT.

At the end of the experimental periods, mice were humanely euthanized using CO_2_. Excised colons were measured then flushed with ice-cold PBS. Tissue was either snap-frozen for biochemical analysis or fixed in 4% paraformaldehyde or 10% phosphate-buffered formalin for 4 to 24 hours, followed by paraffin embedding and hematoxylin and eosin (H&E) staining for histologic analysis. All animal studies were approved by the Institutional Animal Care and Use Committee at Stony Brook University and the University of North Carolina at Chapel Hill.

### Diet-Polarized Fecal Microbiota Transplant

Feces were collected from WT C57BL/6J SPF mice fed BP or PP diets for 2 weeks, anaerobically homogenized, and diluted in prereduced lysogeny broth with 20% glycerol to generate fecal slurries (n = 5–6 donor mice per diet). Each donor FMT was administered to 1 to 2 recipient GF C57BL/6J mice via oral gavage on days 0, 3, 5, 7, and 9 of the experiment. Mice were administered 2% DSS from days 3 to 10 of the experiment and monitored for signs of colitis.

### Inoculation of Defined Consortia

*E coli* LF82,^62^ *E faecalis* OG1RF,^63^ and *R gnavus* ATCC 29149^63^ (EER) were grown under anaerobic conditions in brain-heart infusion medium supplemented with 5 g/L yeast extract, 0.5 g/L L-cysteine, and 5 mg/L hemin (LYH-BHI medium). *A muciniphila* ATCC BAA-835 (AM) was grown under anaerobic conditions in LYH-BHI medium supplemented with 5 g/L porcine gastric mucin. Pure cultures of each strain were grown anaerobically to confluence, then equally mixed to generate EER or EER + AM consortia. GF 129S6/SvEv *Il10*^*−/−*^ mice were colonized with freshly cultured consortia by oral gavage and given a BP diet from the time of colonization. Feces were collected at necropsy, serial diluted, and plated under anaerobic conditions on LYH-BHI medium supplemented with 5 g/L porcine gastric mucin to confirm colonization with consortia members. Representative colonies of each strain identified by colony morphology were picked from plates, 16S PCR–amplified, and Sanger-sequenced (Eton Bioscience) to confirm colonization of appropriate strains in recipient mice.

### Histopathologic Scoring

Colons from mice given DSS were flushed, formalin-fixed, and paraffin-embedded, followed by sectioning and staining with H&E. Scoring of colitis/colonic inflammation was performed based on evaluation of mucosal damage by acute inflammation, by crypt abscess, mucosal architectural distortion, and proportion of the involvement of colon as previously described.^64^ Briefly, epithelial damage by acute inflammation was graded into 4 categories (0–3; 0 = no inflammation; 1 = mild; 2 = moderate; and 3 = severe inflammation with ulceration). Mucosal damage with crypt abscess graded into 2 categories (0 = absent and 1 = present). Extent of acute inflammation was graded into 4 categories (0–3; 0 = no inflammation; 1 = mucosal; 2 = submucosal; and 3 = transmural). Mucosal architectural distortion was graded into 4 categories, which includes the percentage of length of colon involvement (0–3; 0 = normal architecture; 1 = mild/focal or 10%; 2 = moderate/20%–30%; and 3 = severe/>40% of the entire length). Proportion of the total involvement was evaluated by the percentage of length of colon involved by colitis into 4 categories (0–3; 0 = no colitis; 1 = 1%–10% of the total length; 2 = 20%–30%; and 3 = >40% of the total evaluated length of colon). All histologic scoring was performed in a blinded fashion by a board-certified gastrointestinal pathologist (S.A.).

For *Il10*^*−/−*^ experimental colitis studies, histologic inflammation was quantified in ileal, cecal, and colonic (proximal, distal, rectal) tissue segments using a well-validated rubric by blinded histopathology scoring on a scale of 0 to 4 for each segment, as previously described.^32^^,^^64^^,^^65^ Total histology score was calculated by summation of all 5 tissue segment scores for a scale of 0 to 20. Histopathologic scoring was performed blinded to group and experiment.

### Fecal Lipocalin-2 Quantification

Fecal samples were homogenized in PBS with 0.1% Tween 20, incubated at 4 °C for 12 hours, then centrifuged to yield clear supernatant for lipocalin-2 enzyme-linked immunosorbent assay, performed according to the manufacturer’s instructions (DY1857; R&D Systems).^66^

### Measurements of Mucus Thickness and Quality

Segments of harvested colons from mice containing a fecal pellet were preserved in fresh Carnoy’s fixative solution (methanol:chloroform:glacial acetic acid, 60:30:10) then sequentially washed in methanol, ethanol, and xylenes, as previously described.^21^ Tissues were then paraffin-embedded and sectioned (5 μm), followed by staining with Alcian Blue, and mucus thickness was measured in 20 to 50 regions of each colon in a blinded manner using ImageJ software (National Institutes of Health), as previously described.^21^

To determine differences in mucus quality on the surface of the epithelium and within goblet cells, Periodic acid Schiff staining was performed. Stained slides were scanned using an Aperio Image Scope (ScanScope) and used to count the Periodic acid Schiff–positive pixels by the Aperio Color Deconvolution algorithm, as previously described.^21^

### Quantitative Real-Time Polymerase Chain Reaction

For measurements of mammalian gene expression, RNA was isolated from tissues using QIAzol Lysis Reagent (Qiagen) following the manufacturer’s protocol. RNA concentration was measured using a NanoDrop ND-1000 spectrophotometer. Complementary DNA was synthesized using 2000 ng total RNA and reverse transcriptase qScript SuperMix (Quanta). qRT-PCR was performed using specific primers on a QuantStudio 7 Real-Time PCR System (Applied Biosystems) using PowerTrack SYBR Green PCR Master Mix and oligo (dT) primers according to the user’s manual. *Actb* expression was used as internal control for gene expression. The ΔΔCT method was used to calculate relative fold expression. Primer sequences are shown in Table 3.

**Table 3:** Primer Sequences

Gene	Type	Sequence (5′–3′)
Murine targets		
*Actb (beta-actin)*	Forward	GGCTGTATTCCCCTCCATCG
*Actb (beta-actin)*	Reverse	CCAGTTGGTAACAATGCCATGT
*Il1b*	Forward	TGGGCCTCAAAGGAAAGAAT
*Il1b*	Reverse	CAGGCTTGTGCTCTGCTTGT
*Tnfa*	Forward	ACCCTCACACTCAGATCATCTTCTC
*Tnfa*	Reverse	TGAGATCCATGCCGTTGG
Bacterial PCR targets		
*Akkermansia muciniphila*	Akk-16S-F	GATAGTACCACAAGAGGAAGAGACG
*A muciniphila*	Akk-16S-R	TTCCCCCTCCATTACTCTAGTCTC
*Lactobacillus johnsonii*	Ljohn-16S-F	GCCTAGATGATTTTAGTGCTTGCAC
*L johnsonii*	Ljohn-16S-R	AGCAGAACCATCTTTCAAACTCTAGA
*Turicibacter sanguinis*	Turi-16S-F	TGTGACGGTACCTTATGAGAAAGC
*T sanguinis*	Turi-16S-R	GTTGACCAGTTTCCAATGACCCTC
Universal 16s primer	Forward	ACTCCTACGGGAGGCAGCAG
Universal 16s primer	Reverse	ATTACCGCGGCTGCTGG
To amplify EER	Forward	U341F 5′-CCTACGGGRSGCAGCAG-3′
To amplify EER	Reverse	UA1406R 5′-ACGGGCGGTGWGTRCAA-3′

EER, *Escherichia coli*, *Enterococcus faecalis*, and *Ruminococcus gnavus*; PCR, polymerase chain reaction.

For bacterial abundance and bacterial gene expression analysis, DNA was extracted from fecal samples using the QIAamp DNA Fast Stool Mini Kit (Qiagen) per the manufacturer’s instructions. For each sample, 10 ng of DNA was used for qRT-PCR using PowerTrack SYBR green PCR master mix on a QuantStudio 7 PCR Machine. Bacterial primers sequences are shown in Table 3. Relative abundance was calculated by the ΔΔCT method using universal 16S primers as control.

### Bile Acid Analysis

Fecal and ileal samples were dried in a vacuum centrifuge then homogenized in 500 μL of cold 80% methanol. Liver samples were homogenized using the same method but without prior drying. Bacterial supernatants were mixed with cold 80% methanol in a 1:9 ratio. Following homogenization or mixing, samples were centrifuged at 14,000 rcf for 20 minutes at 4 °C, and the supernatant was collected and dried down using a vacuum centrifuge, then stored at −80° C prior to analysis. Dried samples were reconstituted in 50% isopropanol and applied to a Vanquish UHPLC system (Thermo Scientific) coupled to a Q Exactive Orbitrap mass spectrometer (Thermo Scientific) via an Ion Max ion source with a HESI II probe (Thermo Scientific). The mobile phase consisted of buffer A: 60% acetonitrile, 40% water, 10 mM ammonium formate with 0.1% formic acid and buffer B: 90% isopropanol, 10% acetonitrile, 10 mM ammonium formate with 0.1% formic acid. The liquid chromatography gradient was as follows: 0 to 10 minutes, 35% buffer B; 10 to 13 minutes, 35% to 100% buffer B. Mass spectroscopy (MS) data were acquired in negative mode, and a mass scan range of 140 to 1000 m/z at a resolution of 70,000 (at m/z 200), was used. MS data files were processed using XCalibur 4.1 (Thermo Scientific). Identification of BAs was based on accurate masses within 5 ppm and standard retention times. Relative quantitation was performed based on MS signal intensities and normalized to dry weight of fecal/ileal samples or protein concentration of livers. Analysis was performed at the Proteomics & Metabolomics Core Facility of Weill Cornell Medicine.

### Matrix-Assisted Laser Desorption/Ionization Mass Spectrometry Imaging

Tissue preparation and imaging was performed following a previously published method.^67^ Briefly, after harvest, mouse colons were flushed vigorously with ice-cold PBS and processed into Swiss rolls, then embedded in 5% carboxymethylcellulose, and stored at −20 °C. Serial frozen sections were placed onto IntelliSlides (Brucker Daltonics), for paired MALDI imaging and standard H&E staining. MALDI imaging was conducted on a rapifleX mass spectrometer (Bruker Daltonics) within metabolite mass range in negative ion detect mode at 50-μm spatial resolution. The TCA deprotonated ion with a mass-to-charge ratio of 514.284 m/z was detected, and the average intensity of the mass spectrometry signal for TCA was determined using SCiLS Lab software (version 2023c) (Bruker Daltronics).

### 16S Ribosomal RNA Analysis

Frozen fecal samples were shipped to Molecular Research for 16S ribosomal RNA (rRNA) profiling, performed as previously described.^21^ Briefly, DNA was extracted with the Powersoil DNA Kit (Qiagen). For initial 16S-seq studies, the 16S rRNA gene V4 variable region was targeted for PCR amplification, followed by Illumina HiSeq short-read sequencing. Sequencing outputs were processed and taxonomically classified as previously described.^21^ Operational taxonomic units were defined by clustering at 97% similarity (3% divergence) and classified by BLASTn against an RDPII/NCBI–derived database. In subsequent 16S-seq studies, the 16S rRNA amplicon (variable regions 3–4) was sequenced on the Illumina MiSeq platform, processed, and then taxonomically classified using QIIME2^68^ with GreenGenes2^69^ by the University of North Carolina Microbiome Core. Statistical analysis and visualization of PCoA plots and taxonomic barplots were performed with the Shiny application *Plotmicrobiome* (Sun et al; GitHub https://github.com/ssun6/plotmicrobiome). FMT transfer efficiency was quantified at the amplicon sequence variant level by computing Pearson correlation coefficient (*r*) for donor-recipient pairs with counts normalized for sequencing depth with a pseudocount using the following formula as previously described^32^:

$$\log\;10\left(\frac{raw\;OTU\;count\;for\;{sample}_{i}}{total\;sequences\;for\;{sample}_{i}}\;\times \;average\;sequence\;depth\;+\;1\right)$$

### Fluorescence in Situ Hybridization

FISH to detect *A muciniphila* in 4-μm histologic sections of Carnoy’s fixed paraffin-embedded colon tissue was performed as previously described,^21^^,^^70^ using an *A muciniphila*–specific FISH probe (S-SMUC-1437-a-A-20: Cy-3-50-CCTTGCGGTTGGCTTCAGAT-30) in combination with non-EUB-338 (6-FAM-5′-ACTCCTACGGGAGGCAGC), and positive (AM) and negative (*E coli* DH5α, *Proteus vulgaris, Klebsiella pneumoniae, Streptococcus equi, Streptococcus bovis*) controls.^21^^,^^70^

### In Vitro Bacterial Culture and Treatment

*L johnsonii* BAA-3147 and *A muciniphila* BAA-835 from frozen stocks were cultured overnight in MRS or LYH-BHI + 0.5% mucin, respectively, the day before the experiments. *A muciniphila* BAA-835 was cultured in an anaerobic chamber (Coy Laboratory) with mixed gas (90% N_2_, 5% CO_2_ and 5% H_2_; 37 ^o^C), and *L johnsonii* BAA-3147 was cultured under aerobic conditions (37 ^o^C with shaking at 150 rpm). The overnight cultures were diluted with fresh media with or without 1 mM taurocholate to AM (once at optical density of 600 nm of 1–1.1) and to *L johnsonii* (once at optical density of 600 nm of 0.7–0.8). The diluted cultures were immediately distributed into 2 sets of tubes for 0 and 24-hour timepoints, each in triplicate. The time 0-hour samples were transferred to ice immediately to prevent bacterial activity on taurocholate. Optical densities were measured at each time point for normalization. The cultures were then centrifuged at 3000 rpm for 20 minutes at 4 ^o^C, and the supernatants were filtered through a 0.2-μm syringe filter and kept at −80^o^C until BA measurements.

### Statistical Analysis and Power Calculations

Estimated sample size for mouse experiments was calculated based on the primary diet effect using a 1-way analysis of variance (fixed effects, omnibus) in G∗Power 3.1 using the standard thresholds for α = .05 and power (1-β) = .8. Effect size was estimated using data from prior studies performed in the same mouse model systems. DSS studies were performed with a single sex because DSS colitis severity is sex-dependent. For DSS studies with a single sex and 5 diet groups, the DAI score was used to perform power analyses, and the effect size f (Cohen’s f) was estimated to be 0.7 based on historical means and standard deviations from preliminary studies by our group (unpublished data). For a DAI effect size f = 0.7, with α = .05, power (1-β) = .8, with 5 groups, the required total sample size was 30 (6 mice per group × 5 groups). For ex-GF *Il10*^*−/−*^ mouse studies including both sexes and 5 diet groups, the total histology score was used to perform power analyses, and the effect size f was estimated to be 0.9 based on historical means and standard deviations from preliminary studies by our group (unpublished data). For a histology score effect size f = 0.9, with α = .05, power (1-β) = .8, with 5 groups, the required total sample size was 25 (5 mice per group × 5 groups). Per National Institutes of Health guidelines on sex as a biological variable, equal numbers of male and female mice were included in each group (5 males and 5 females per group) for ex-GF *Il10*^*−/−*^ studies. Due to breeding capacity limitations, in some studies, experiments were pooled for statistical analysis to achieve desired power. Each study was powered to detect the main diet effect (α = .05, power (1-β) = .8) using the primary end point for that model (DAI score for DSS; histology score for ex-GF *Il10*^*−/−*^), and analyses of sex-related differences, including sex × diet interaction, were considered exploratory/secondary. In the initial 5-group ex-GF *Il10*^*−/−*^ study, due to non-study–related attrition, some per-group numbers of mice were 7 to 9; however, because the observed effect size f was 1.5 (larger than expected), post hoc power analysis achieved a power (1-β) > .95. For later ex-GF *Il10*^*−/−*^ studies comparing 2 or 3 groups, the same per group sample size was used (5 males and 5 females per group), which with effect size f = 1.5 (observed from 5-group study) and α = .05 achieved power (1-β) > .95 in post-hoc power analysis (G∗Power 3.1, 1-way analysis of variance, fixed effects).

For the comparison of DAI across different mice groups, linear mixed-effects models for longitudinal data analysis were used. Model assumption was confirmed using residual diagnosis. Time (in days) was treated as a continuous variable to assess the trend in DAI. An interaction term between diet group and time was used to compare the trends across groups. The covariance structure used to model the correlated longitudinal measurements from the same mice was unstructured, selected based on Akaike Information Criterion from a set of possible structures. For the comparison of numeric values observed or calculated at the end of experiments, Wilcoxon rank-sum tests or simple linear regression models were used. These analyses were applied to the following variables: % body weight change, histology score, gene expression, colon length, bacterial abundance, mucus thickness, mucus quality, and BA ratios. A *P* value less than .05 was considered statistically significant, and analyses were performed using SAS 9.4 (SAS Institute Inc).

Statistical analysis of BA levels was conducted by filtering data to remove BAs with >50% missing values. Normalization of raw metabolite levels was performed by probabilistic quotient normalization followed by log2-transformation and imputation of missing values by k-nearest neighbor. BA differences were quantified by log2 fold change from the PP diet group. Linear models with diet as the independent variable were used to calculate statistical significance. The Benjamini–Hochberg method with a false discovery rate cutoff of 0.2 was used for multiple hypothesis correction. Analyses were performed with the maplet package (version 1.2.1) in R (version 4.3.2).^71^

Sex-, experiment-, and cage-effects were tested using linear mixed-effects models implemented in R with the lmer function in the lme4 package (version 1.1.37). The response variables were fecal lipocalin-2 level, total histology score, or % weight change; diet, sex, and experiment number were fixed effects, and cage membership was a random effect (https://github.com/simonmgray/dietary_protein_source_mediates_experimental_colitis).
